# Obesity Is Mediated by Differential Aryl Hydrocarbon Receptor Signaling in Mice Fed a Western Diet

**DOI:** 10.1289/ehp.1205003

**Published:** 2012-05-18

**Authors:** Joanna S. Kerley-Hamilton, Heidi W. Trask, Christian J.A. Ridley, Eric DuFour, Carol S. Ringelberg, Nilufer Nurinova, Diandra Wong, Karen L. Moodie, Samantha L. Shipman, Jason H. Moore, Murray Korc, Nicholas W. Shworak, Craig R. Tomlinson

**Affiliations:** 1 Dartmouth-Hitchcock Medical Center, Lebanon, Norris Cotton Cancer Center, Lebanon, New Hampshire, USA; 2 Department of Biology and Biochemistry, University of Bath, Bath, United Kingdom; 3 Department of Genetics, Dartmouth Medical School, Hanover, New Hampshire, USA; 4 Tennessee Technological University, Cookeville, Tennessee, USA; 5 Department of Medicine, and; 6 Department of Pharmacology and Toxicology, Dartmouth-Hitchcock Medical Center, Lebanon, New Hampshire, USA

**Keywords:** aryl hydrocarbon receptor, gene–environment interaction, liver, mRNA, miRNA, obesity, Western diet

## Abstract

Background: Obesity is a growing worldwide problem with genetic and environmental causes, and it is an underlying basis for many diseases. Studies have shown that the toxicant-activated aryl hydrocarbon receptor (AHR) may disrupt fat metabolism and contribute to obesity. The AHR is a nuclear receptor/transcription factor that is best known for responding to environmental toxicant exposures to induce a battery of xenobiotic-metabolizing genes.

Objectives: The intent of the work reported here was to test more directly the role of the AHR in obesity and fat metabolism in lieu of exogenous toxicants.

Methods: We used two congenic mouse models that differ at the *Ahr* gene and encode AHRs with a 10-fold difference in signaling activity. The two mouse strains were fed either a low-fat (regular) diet or a high-fat (Western) diet.

Results: The Western diet differentially affected body size, body fat:body mass ratios, liver size and liver metabolism, and liver mRNA and miRNA profiles. The regular diet had no significant differential effects.

Conclusions: The results suggest that the AHR plays a large and broad role in obesity and associated complications, and importantly, may provide a simple and effective therapeutic strategy to combat obesity, heart disease, and other obesity-associated illnesses.

It has been estimated that 25–70% of the underlying basis for obesity is gene based (Cardon et al. 1994; Stunkard et al. 1986); thus, environmental factors are a major contributor with 30–75% (Baillie-Hamilton 2002). One of the accepted environmental causes for the worldwide rise in obesity and associated problems is the increased consumption of the high-calorie, high-fat, low-fiber Western diet. A biological entity that tightly links genes and the environment is a nuclear receptor best known for its role in xenobiotic metabolism: the aryl hydrocarbon receptor (AHR). AHR is a ligand-activated nuclear receptor/transcription factor that regulates genes involved in toxicant metabolism and provides a major defense to environmental exposures. AHR signaling is also involved in a number of essential nonxenobiotic biological and developmental pathways (Fernandez-Salguero et al. 1995). Upon ligand binding, the AHR translocates to the nucleus, where it complexes with the AHR nuclear translocator (ARNT). The AHR/ARNT heterodimer regulates the transcription of genes in the cytochrome P450 *Cyp1* family, some phase II detoxification genes, and thousands of other genes (Trask et al. 2009), including the gene expression of other nuclear receptors relevant to obesity [e.g., *Ppara* (peroxisome proliferator–activated receptor-α)] (Wang et al. 2011). The AHR is also activated by dietary components such as fats and fat derivatives (McMillan and Bradfield 2007), and there is evidence linking the activated AHR to major diseases, including obesity (La Merrill et al. 2009).

Although several studies have examined the relationship between the AHR and fat metabolism using a model system comparing functional AHR signaling to one that is AHR deficient, none have examined the consequences resulting from different levels of AHR signaling activity. To identify a possible role for the AHR in obesity, we used two mouse models that differ at the *Ahr* gene (Figure 1A). The two strains were C57BL/6 (B6 strain), which naturally bears the high-affinity AHR encoded by the *Ahr*^b1^ allele, and the congenic C57BL/6.D2 (B6.D2 strain), which bears the low-affinity AHR encoded by the *Ahr*^d^ allele naturally found in the DBA/2 mouse strain. The two *Ahr* alleles encode AHRs that differ by approximately 10-fold in ligand binding affinity, as well as gene induction and gene expression levels, including that of the *Cyp1a1* and *Cyp1b1* xenobiotic genes (Thomas et al. 2002). A distinct advantage of using the B6 and B6.D2 mouse models is that by virtue of the integral role the AHR plays in response to endogenous and environmental agents, any corresponding differences observed in disease states, gene expression profiles, and affected signaling pathways are due to the differing capacities of the corresponding AHRs.

**Figure 1:**
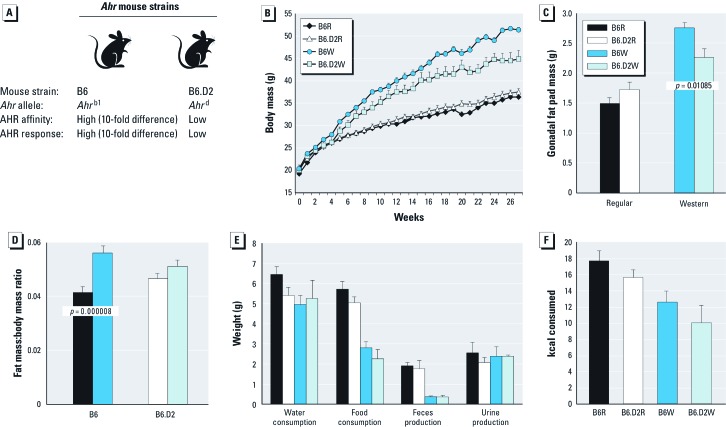
Effect of diet on male B6 and B6.D2 mice. (*A*) B6 and B6.D2 mouse strains. (*B*) Body mass of mice fed the regular diet or Western diet for 28 weeks (*n* = 8 mice/group). Gonadal fat pad mass (*C*) and gonadal fat pad mass:body mass ratio (*D*) of mice fed the diets for 28 weeks (*n* = 8 mice/group). (*E*) Consumption and excretion amounts (*n* = 3 mice/group) at week 20. (*F*) Consumed kilocalories during a 48-hr period in week 20 (*n* = 3 mice/group). Values are mean ± SE.

There have been hints that the AHR may be a participant in the regulation of fat metabolism and obesity (Arsenescu et al. 2008; Kerley-Hamilton et al. 2012). The intent of our study was to test more directly the role of the AHR in obesity and fat metabolism, but without exposure to exogenous toxicants. We tested the hypothesis that differential AHR signaling activity differentially affects body mass and liver metabolism. Using the B6 and B6.D2 mouse models, we found that AHR signaling activated to different levels by a Western diet drastically affected relative fat mass, liver physiology, and liver gene expression.

## Materials and Methods

*Materials.* The low-fat (regular) mouse chow (catalog no. 2018; 3.1 kcal/g; 24% kcal protein, 58% kcal carbohydrates, 18% kcal fat) and the high-fat (Western) mouse chow (catalog no. TD.88137; 4.5 kcal/g; 15% kcal protein, 43% kcal carbohydrates, 42% kcal fat) were purchased from Harlan Laboratories (Madison, WI). The Western diet contains no detectable phytoestrogens or xenobiotics (personal communication, Harlan Laboratories).

*Mice.* We obtained male C57BL/6J and B6.D2N-*Ahr*^d^/J mice (stock numbers 000664 and 002921, respectively) from The Jackson Laboratory (Bar Harbor, ME), where they are maintained. The C57BL/6 mouse (B6) has the high-affinity AHR (*Ahr*^b1^ allele), and the congenic C57BL/6.D2 mouse strain (B6.D2) has the low-affinity AHR (*Ahr*^d^ allele) (Hofstetter et al. 2007) (Figure 1). *Ahr*^b1^ is the naturally occurring allele in C57BL/6J mice. The *Ahr*^d^ allele, from the DBA/2J mouse strain, was introgressed into the C57BL/6J background for > 40 generations. B6.D2 mice have a genomic insert on chromosome 12 from the DBA/2J mouse genome; this insert spans 35.4–41.0 Mbp and contains 15 genes. Of these 15 genes, only the *Ahr* and *Zfp277* genes contain nonsynonymous single nucleotide polymorphisms (Hofstetter et al. 2007). The *Ahr* allele for each mouse was confirmed by genotyping (Song et al. 2004).

Beginning at 5 weeks of age, the B6 and B6.D2 male mice (*n* = 8 mice/group) were fed the regular diet (low fat; B6R and B6.D2R, respectively) or the Western diet (high fat; B6W and B6.D2W, respectively) for 28 weeks. Body weight of each animal was recorded weekly. We examined eating behavior of the mice at week 20 by individually housing three mice from each experimental group in mouse metabolic cages for 96 hr to acclimate. Water and chow intake and feces and urine output were then measured over the course of the next 48 hr. At the end of the 28-week period, all mice were sacrificed. To determine white fat accumulation, we dissected and weighed gonadal fat pads; values are reported as gonadal fat pad mass:body mass (*n* = 8 mice/experimental group). Blood and liver tissue were also collected for analysis. All animals were treated humanely and with regard for alleviation of suffering.

*Histology.* Sections (~ 5 mm thickness) from formalin-fixed, paraffin-embedded liver samples were stained with hematoxylin and eosin (H&E). The histology procedures were carried out by the Pathology Shared Resource at Dartmouth-Hitchcock Medical Center. The stained slides were examined at 200× magnification using a Nikon Eclipse 80i microscope (Nikon Instruments Inc., Melville, NY). Images were generated using identical settings with a MicroPublisher 5.0 real-time viewing camera (QImaging, Surrey, British Columbia, Canada). The images were analyzed using ImageJ (National Institutes of Health, Bethesda, MD). We examined 10 different fields per liver section from four mice in each experimental group to ensure that the images were representative of the livers for a given group. Total vacuole area in a given microscopic field of vision was defined as the total amount of (undefined) light units captured from binary images at a brightness threshold set at 200, the setting at which the binary image was most similar in contrast to that of the corresponding color image.

*Plasma chemistry.* At sacrifice, plasma was obtained from blood samples by centrifugation and stored at –80°C. Plasma was analyzed for alanine aminotransferase (ALT), aspartate aminotransferase (AST), alkaline phosphatase (ALP), total protein, and total cholesterol by the Serology/Clinical Pathology Division of Charles River Laboratory (Wilmington, MA). Because the available plasma volume for some samples was < 300 µL, plasma chemistry measurements could not be obtained for all samples.

*RNA purification.* Livers were sliced into smaller pieces and homogenized in TRIzol Reagent (Invitrogen Corp., Carlsbad, CA). RNA purity, quantity, and quality were determined using a NanoDrop ND-1000 spectrophotometer (Thermo Scientific, Waltham, MA) and an Agilent 2100 Bioanalyzer (Agilent Technologies, Santa Clara, CA) (Wang et al. 2006).

*Microarrays.* The mRNA and miRNA gene expression microarray experiments were carried out by the Dartmouth Genomics and Microarray Laboratory. For mRNA profiling, we used the MouseRef-8 v2.0 Expression BeadChip array (Illumina, San Diego, CA), with approximately 25,600 annotated RefSeq transcripts covering 19,100 unique mouse genes. Approximately 0.5 mg of total RNA per mouse liver was labeled for each bead array as described by Thornley et al. (2011). The bead arrays were scanned with an Illumina 500GX scanner and processed with BeadScan software (both from Illumina). Differential levels of miRNA and other noncoding RNAs were determined using the Affymetrix GeneChip miRNA 2.0 Array (Affymetrix, Santa Clara, CA), which contains a 15,644 probe set to all known miRNAs. Approximately 0.5 mg of total RNA per mouse liver was labeled for each array as described in the manufacturer’s instructions. The arrays were scanned with an Affymetrix GeneChip Scanner 3000 and processed with Affymetrix miRNA QCTool software.

*Data analysis.* Four biological replicates per experimental condition were included in the microarray studies. We performed quantile normalization (Smyth and Speed 2003) without background correction to preprocess the image files generated by the Illumina software. Analyses were performed using BRB-Array Tools Version 4.2.1 (Wright and Simon 2003). Genes that were differentially expressed among classes were identified using a random-variance *t*-test. Genes were considered statistically significant at *p* ≤ 0.05. The data were also analyzed using Pathway Studio (Ariadne Inc., Rockville, MD) to generate gene lists with designated false discovery rates (FDR) (Reiner et al. 2003). Statistically significant, differentially expressed genes were annotated with functional assignments to help determine category enrichment using the biological process (BP-FAT) branch of the Gene Ontology (GO) database (Ashburner et al. 2000) via the DAVID program provided by the National Institute of Allergy and Infectious Diseases (Huang et al. 2009). Venn diagrams of the statistical results were constructed using GeneVenn software (Pirooznia et al. 2007). *p*-Values were calculated using paired Student’s *t*-test.

*Quantitative polymerase chain reaction (qPCR).* To verify microarray results, we performed qPCR analysis using SYBR green and designed primers [see Supplemental Material, Table S1 (http://dx.doi.org/10.1289/ehp.1205003)] as described by Schwanekamp et al. (2006). Approximately 2 mg of total RNA (the same RNA used for the microarrays) served as a template for cDNA synthesis. The qPCR reactions were performed on a DNA Engine Opticon Monitor System using version 3.1 software (BioRad, Hercules, CA) set at 40 cycles. Agarose gel electrophoresis showed that each PCR produced a single band of the predicted size. Assays to determine DNA contamination were carried out by omitting reverse transcriptase from the reactions. The qPCR results confirmed the microarray results (see Supplemental Material, Table S2).

## Results

*Differential AHR signaling and obesity.* We used two congenic mouse models (Figure 1A) that encode AHRs that differ by 10-fold in signaling activity (Poland and Glover 1980). Male mice from B6 and B6.D2 mouse strains were placed into two diet groups (*n* = 8/group) and fed low-fat regular chow or high-fat Western chow for 28 weeks beginning at 5 weeks of age. By 17 weeks, B6W mice had significantly greater body mass than did the B6.D2W mice (Figure 1B); at the conclusion of the study (week 28), B6W mice were > 16% larger than their B6.D2 counterparts [see Supplemental Material, Tables S3 and S4 (http://dx.doi.org/10.1289/ehp.1205003)].

The increased body mass observed in B6 mice compared with B6.D2 mice could be due to an overall proportional increase in body size rather than an increased relative accumulation of body fat. The gonadal fat pad mass:body mass ratio highly correlates to the overall body white fat mass:body body mass ratio (Rogers and Webb 1980). B6W mice had significantly greater gonadal fat pad mass than B6.D2W mice (Figure 1C). B6W mice had a significantly greater fat mass:body body mass ratio than B6R mice, whereas B6.D2R and B6.D2W mice were not statistically different (Figure 1D).

To determine whether the significant differences in body mass observed between the two mouse strains on the Western diet were due to metabolic differences rather than differences in behavioral eating habits, we measured food and water intake and urine and feces production in three mice from each group at week 20. Although there were significant differences in the amount of Western and regular chow consumed and the amount of feces generated (*p* ≤ 0.05; Figure 1E), we observed no significant differences between the two mouse strains in any of the measured parameters. Furthermore, caloric intake for the mice (Figure 1F) was calculated based on the kilocalories per gram of regular (3.1 kcal/g) and Western (4.5 kcal/g) chows times the grams of chow consumed (Figure 1E). We observed no significant differences in consumed calories between the B6 and B6.D2 mice on either regular diet (17.7 kcal and 15.6 kcal, respectively) or Western diet (12.6 kcal and 10.1 kcal, respectively), nor did we see any significant differences in caloric intake between diets in a given strain. Thus, the difference in body mass between the B6W and B6.D2W mice was not due to differences in consumption and excretion behaviors. These data and the results above suggest that there is an AHR-dependent metabolic basis for the significant increase in body mass and relative fat amounts observed in the B6W and B6.D2W mice.

**Figure 2:**
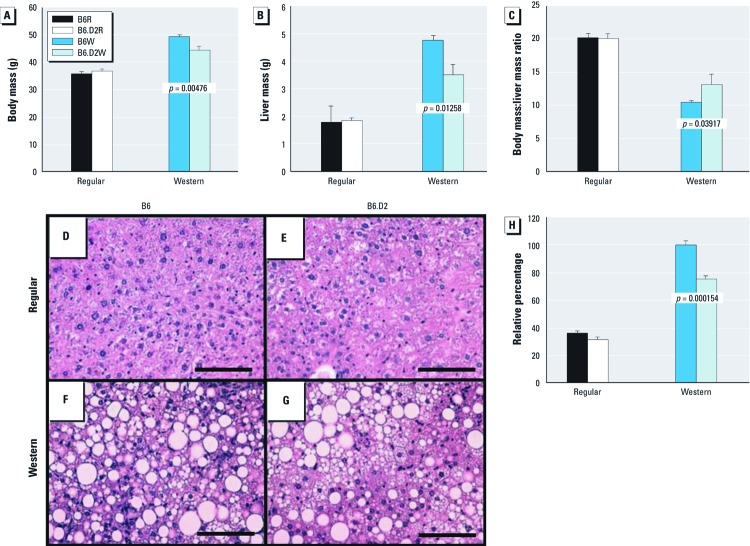
Effect of diet on liver size and on and fat content in the livers of male B6 and B6.D2 mice. Body mass (*A*), liver mass (*B*), and body mass:liver mass ratio (*C*) of B6 and B6.D2 mice (*n* = 8 mice/group) fed regular diet or Western diet for 28 weeks. (*D–G*) Photomicrographs of H&E-stained liver sections (200× magnification; bar = 100 mm) from B6 mouse fed regular diet (*D*), B6.D2 mouse fed regular diet (*E*), B6 mouse fed Western diet (*F*), and B6.D2 mouse fed Western diet (*G*). (*H*) Total vacuole area in liver per 10 fields of vision for (*n* = 4/group). For *A–C* and *H*, values are mean ± SE.

*Liver size and metabolism.* The liver is the primary site of dietary fat metabolism and regulates fat levels in the blood. Several findings led us to conclude that differential AHR activity had a large impact on liver growth and metabolism. For both mouse strains, the Western chow not only had a major impact on body mass after the 28-week diet regimen (Figure 2A) but also on liver mass (Figure 2B): All mice fed the Western diet had an approximately 2-fold increase in liver mass relative to body mass compared with mice fed regular chow (Figure 2C). However, the impact of the Western diet on liver size was greater for B6 mice, in that they had significantly larger livers and significantly smaller body mass:liver mass ratios than did B6.D2W mice.

The hepatomegaly observed in the mice fed the Western diet is reminiscent of nonalcoholic fatty liver disease, which is most often caused by the accumulation of fat in the liver in obese individuals (Adams et al. 2005). We investigated whether there was differential fat accumulation in mice fed Western versus regular chow and whether there were genotypic differences for fat accumulation between strains for each diet. Liver sections were stained with H&E, which can reveal the presence of fat storage vesicles. We observed no discernible fat vesicles in B6R and B6.D2R mice (Figure 2D,E) and no significant difference in fat vesicle volume (Figure 2H). However, both mouse strains fed Western diet had a significantly greater volume of fat vesicles than the control groups. Furthermore, B6W mice had a significantly greater volume of fat storage vesicles than did B6.D2W mice (*p* = 1.54 × 10^–8^) (Figure 2F–H). The results suggest that the different levels of hepatomegaly observed in the two mouse strains fed Western diet is due to AHR-dependent differential fat accumulation in hepatocytes.

**Figure 3:**
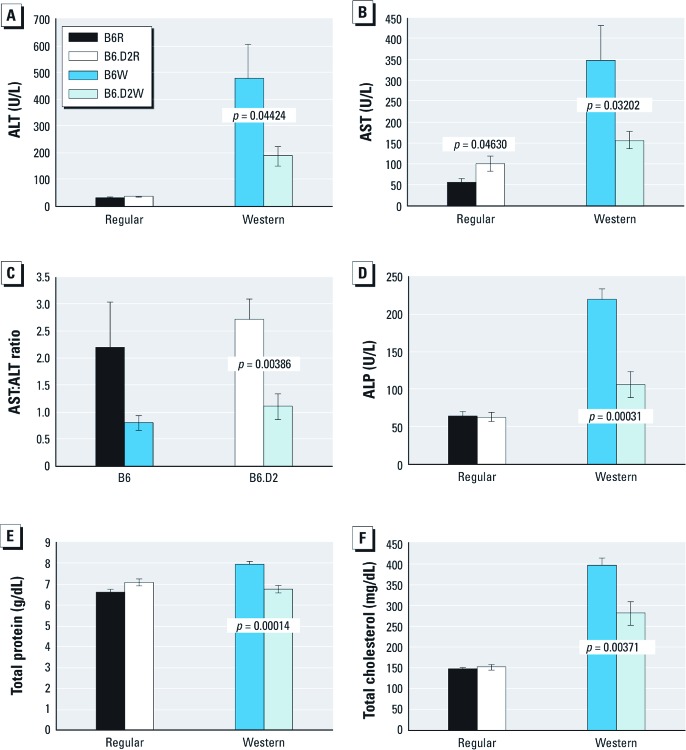
Levels of liver damage markers and cholesterol in male B6 and B6.D2 mice fed regular diet or Western diet for 28 weeks. (*A*) ALT (reference range, 27.3–115.3). (*B*) AST (reference range, 45.0–386.1). (*C*) AST/ALT ratio. (*D*) ALP (reference range, 65.5–272.6). (*E*) Total protein (reference range, 4.6–6.9). (*F*) Total cholesterol (reference range, 74.0–167.0). Values are mean ± SE (*n* = 8 mice/group).

ALT levels rise dramatically in acute liver damage, whereas the plasma level of AST is an indicator of hepatic and extrahepatic tissue damage. B6W mice had significantly higher plasma levels of both AST and ALT than B6.D2W mice (Figure 3A,B). However, we observed somewhat less disparity in AST:ALT ratios in B6W mice compared with B6R mice (no significant difference); however, in B6.D2 mice, there was a significant difference between animals fed the two diets (*p* = 0.00386) (Figure 3C). These results suggest that among mice fed Western diet, B6 mice suffered relatively higher levels of extrahepatic damage (e.g., possible kidney, cardiac muscle, and/or skeletal muscle injury) than did B6.D2 mice.

B6W mice had significantly elevated plasma levels of ALP, total protein, and total cholesterol compared with B6.D2W mice (Figure 3D–F). An increased level of ALP (Figure 3D) is another measure of a number of liver anomalies, including obesity (Golik et al. 1991). Increased total protein levels (Figure 3E) can be associated with liver disease but often remain in the normal range (4.6–6.9 g/dL), typically due to a decrease in plasma albumin concentration and a concomitant increase of plasma globulin levels, including ALT, AST, and ALP. However, we observed no significant differences in plasma albumin levels between B6 and B6.D2 mice (data not shown), and we surmised that the normal total proteins levels observed in B6 mice was due primarily to the increased globulin levels. Increased plasma levels of total cholesterol (Figure 3F) are associated with the chronic consumption of fatty diets (Turley et al. 1998).

**Figure 4:**
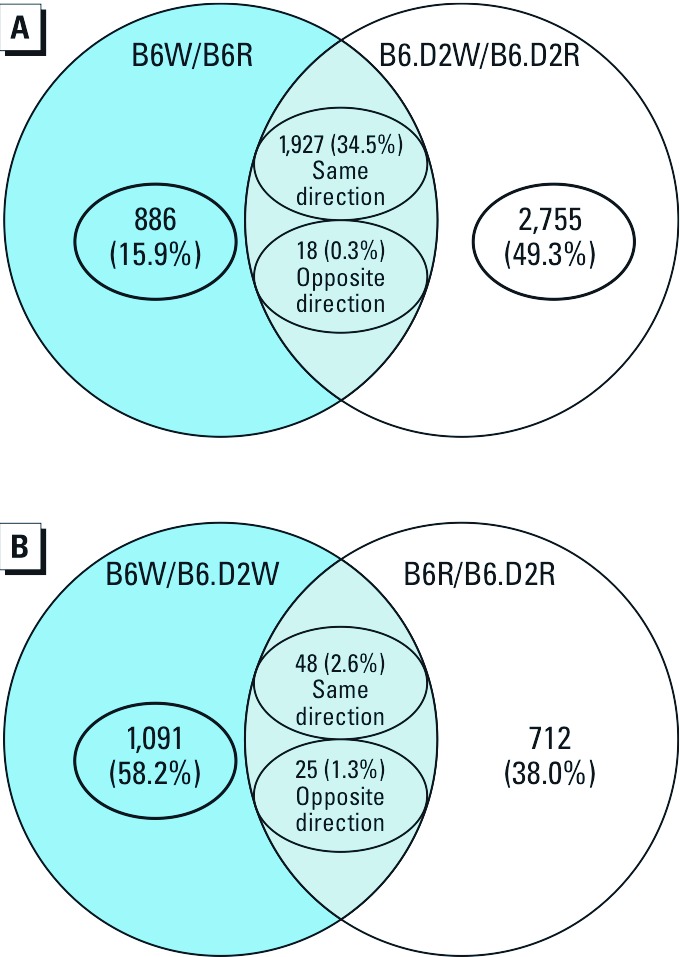
Shared and uniquely differentially expressed genes from B6 and B6.D2 mice fed regular or Western diets by micro­array analysis (*n* = 4 mice/group). Venn diagrams display the number of differentially expressed genes from the effect of diet on *Ahr* genotype (*A*) and the effect of *Ahr* genotype on diet (*B*).

*mRNA profiles of liver.* To determine the effect of diet on a given *Ahr* genotype, we compared the mRNA levels from livers of B6W and B6.D2W mice with those from mice of the same strain fed regular diet [Figure 4A; see also Supplemental Material, Table S5 (http://dx.doi.org/10.1289/ehp.1205003)]. The mRNA levels of some genes known to be involved in obesity, lipid and sterol metabolism, and inflammation—many of which contained AHR promoter response elements (REs) (Sun et al. 2004)—were affected by Western diet in B6 and B6.D2 mice: *ApoA4* (15.9-fold and 10.2-fold), which is involved in innate immunity and fat localization (Shen et al. 2007), and *Hsd3b5* (↓0.03-fold, ↓0.03-fold), a gene associated with hepatic steatosis (Guillen et al. 2009). Although a given gene may contain an AHR RE(s), the element(s) may or may not be playing a regulatory role. Some potentially key genes uniquely and differentially expressed in the B6W/B6R comparison group (Table 1) included multiple mRNA forms of *Insig1* (insulin induced gene 1; ↓0.40-fold and 0.37-fold; 12 AHR REs); INSIG1 is a key regulator in cholesterol metabolism (Kast-Woelbern et al. 2004). In addition to the AHR, *Insig1* is regulated by multiple nuclear receptors including PPARa (15 AHR REs), CAR (constitutive androstane receptor; 2 AHR REs), and PXR (pregnane X receptor). Some uniquely differentially expressed genes in the B6.D2W/B6.D2R mice (Table 1) included *Hamp2* (hepcidin antimicrobial peptide 2; 9.1-fold), which has a role in iron metabolism and SMAD phosphorylation (Kautz et al. 2008), and *Creld2* (cysteine-rich with EGF-like domains 2; ↓0.3-fold), an endoplasmic reticulum stress-induced gene (Oh-hashi et al. 2009). Cellular pathways expressed uniquely in B6W mice compared with B6R mice were associated with inflammation (Table 1); and genes and pathways unique to B6.D2 mice dealt more with cellular housekeeping functions, including protein localization and DNA repair (Table 1).

**Table 1:** The 20 genes with the greatest change in differential mRNA expression (*p* ≤ 0.05) and associated cellular pathways (FDR ≤ 1.0) in B6W/B6R, B6.D2W/B6.D2R, and B6W/B6.D2W comparisons.

Gene	Fold change	No. of AHR REs	Pathways
Unique to B6W/B6R						Unique to B6W/B6R
Mt1		4.17		0		GO:0002026 regulation of the force of heart contraction
C1qb		3.03		5		GO:0006954 inflammatory response
H2-Ab1		2.78		0		GO:0009611 response to wounding
Tnfrsf12a		2.63		10		
Chac1		2.63		0		
Saa1		2.56		0		
C1qc		2.38		0		
Hspb1		2.33		0		
Plk3		2.27		9		
Lgmn		2.22		2		
Ctsa		0.51		0		
Slc38a4		0.51		2		
Insig1		0.51		12		
Sc5d		0.48		5		
Aldoc		0.47		0		
LOC100040592		0.46		0		
Ppp1r3c		0.43		0		
Sucnr1		0.42		0		
Insig1		0.40		12		
Insig1		0.37		12		
Unique to B6.D2W/B6.D2R						Unique to B6.D2W/B6.D2R
Hamp2		9.09		0		GO:0046907 intracellular transport
Spon2		3.33		3		GO:0045184 establishment of protein localization
8430408G22Rik		3.13		4		GO:0015031 protein transport
Lip1		2.94		2		GO:0033554 cellular response to stress
4930572J05Rik		2.94		0		GO:0008104 protein localization
Gsta2		2.86		0		GO:0006974 response to DNA damage stimulus
Gstm2		2.86		3		GO:0009057 macromolecule catabolic process
Lbh		2.78		0		GO:0044265 cellular macromolecule catabolic process
Gstm2		2.70		3		GO:0034613 cellular protein localization
Srebf1		2.63		7		GO:0006259 DNA metabolic process
Hsp105		0.44		8		GO:0070727 cellular macromolecule localization
Scara5		0.43		0		GO:0030163 protein catabolic process
G6pc		0.42		6		GO:0006886 intracellular protein transport
Lpin1		0.40		12		GO:0006281 DNA repair
Hamp		0.40		2		GO:0051186 cofactor metabolic process
Ccbl2		0.34		0		GO:0006396 RNA processing
Acot1		0.33		0		GO:0055114 oxidation reduction
Eif4ebp3		0.29		0		GO:0044257 cellular protein catabolic process
Creld2		0.28		0		GO:0051603 proteolysis in cellular protein catabolism
Creld2		0.27		0		GO:0006888 ER to Golgi vesicle-mediated transport
Unique to B6W/B6.D2W						Unique to B6W/B6.D2W
Cyp2d26		42.02		2		GO:0055114 oxidation reduction
Gadd45g		2.50		12		GO:0051186 cofactor metabolic process
Bhmt		2.09		7		GO:0033554 cellular response to stress
Gadd45g		2.05		12		GO:0008610 lipid biosynthetic process
Bax		1.83		0		GO:0006732 coenzyme metabolic process
Gpd1		1.75		7		GO:0008202 steroid metabolic process
Clec4f		1.72		0		GO:0001568 blood vessel development
Bst2		1.69		0		GO:0006974 response to DNA damage stimulus
Hmox1		1.60		4		GO:0001944 vasculature development
Ccl4		1.57		4		
Cyp17a1		0.59		3		
Gnat1		0.59		0		
Gpam		0.58		4		
Sc4mol		0.58		6		
Pcsk9		0.57		6		
Spp1		0.56		3		
Sqle		0.52		8		
Esm1		0.50		0		
Insig1		0.50		12		
Insig1		0.46		12		
ER, endoplasmic reticulum.						

In addition to determining the effect of diet on the *Ahr* genotypes, we wanted to examine the effect of *Ahr* genotype for each diet (Figure 4B). Some potentially important genes expressed uniquely in the B6W/B6.D2W comparison, in which some contained AHR REs (Table 1), included *Cyp2d26*, *Gadd45g*, *Bhmt*, and *Sqle*, as well as *Insig1* (↓0.46-fold; 12 AHR REs). *Cyp2d26* (42-fold; 2 AHR REs) is a candidate gene for the regulation of triglyceride levels (Leduc et al. 2011); *Gadd45g* (2.50-fold; 12 AHR REs) encodes a protein that functions in T-cell production (Chi et al. 2004); the product of *Bhmt* (2.09-fold; 7 AHR REs) is associated with liver steatosis and injury and protects hepatocytes from endoplasmic reticulum stress and excess lipid accumulation (Ji et al. 2007); and the obesity-associated *Sqle* (↓0.52-fold; 8 AHR REs) gene encodes a protein that carries out a step in cholesterol biosynthesis (Yamamoto and Bloch 1970). Genes relevant to obesity expressed uniquely in the B6R/B6.D2R comparison included *Ppp1r3c* and *Elovl3*. The *Ppp1r3c* (2.3-fold) gene product is involved in glycogen storage in adipocytes (Greenberg et al. 2006), and loss of the *Elovl3* (2.2-fold) gene in mice causes reduced adiponectin levels, inhibition of adipose tissue expansion, and resistance to diet-induced obesity (Zadravec et al. 2010). The major biological pathways affected in B6W versus B6.D2W mice were involved in fat metabolism and synthesis, vasculature, and sterol metabolism (Table 1).

*miRNA profiles of liver.* Studies have shown an important role for miRNAs in fat metabolism (McGregor and Choi 2011). All statistically significant (*p* ≤ 0.05), differentially expressed miRNA levels from B6 and B6.D2 mice fed either diet are listed in Supplemental Material, Table S6 (http://dx.doi.org/10.1289/ehp.1205003). Those differentially expressed miRNAs with a fold-change > 2 and those with roles known to be associated with obesity, nonalcoholic fatty liver disease, and adipogenesis (e.g., mmu-miR-130b and mmu-miR-132) are shown in Supplemental Material, Table S7. However, because most of the differentially expressed miRNAs have not been described previously as playing a role in obesity, they deserve further scrutiny.

## Discussion

*AHR signaling and obesity.* There are four well-characterized *Ahr* allelic variants in mice, of which there is a 10-fold difference in the affinity for the AHR ligand between the most-responsive allele (*Ahr*^b1^ in the B6 mouse) and least-responsive allele (*Ahr*^d^ in the B6.D2 mouse) (Poland et al. 1994). The 10-fold difference in affinity corresponds to a 10-fold difference in AHR activity. The difference in affinity is primarily due to the replacement of an alanine at position 375 (high-affinity *Ahr*^b1^) with a valine (low-affinity *Ahr*^d^) (Ema et al. 1994). The different AHR affinities were determined using predominantly xenobiotic ligands, and it is possible that any putative endogenous ligand(s) in the Western chow may have had similar AHR binding characteristics in the B6 and B6.D2 mice.

Nonetheless, the results from the mouse model studies should be translatable to humans because the affinity and responsiveness of the mouse *Ahr*^d^ gene is similar to that of the human *Ahr* gene: The human AHR has a valine at the position equivalent to position 375 in the mouse AHR (Moriguchi et al. 2003). In humans, there is no evidence that the several identified forms of the AHR have different ligand binding affinities. However, epidemiological studies have shown an association between various polymorphic forms of the AHR and cancer (Harper et al. 2002), but none have shown an association to obesity. A large-scale epidemiological study to investigate a possible association between the human AHR and obesity has not been conducted.

Experimental work in mice has revealed a possible link between AHR signaling and obesity and fat metabolism. Toxicant-induced AHR signaling inhibited lipid synthesis and adipocyte differentiation, and the loss of AHR activity caused an increase in triglyceride synthesis (Alexander et al. 1998). Constitutive AHR signaling in mouse liver and intestine caused *a*) an increase in the levels and activity of CD36 (a cell-surface fatty acid receptor and translocase in which the *Cd36* gene is a transcriptional target of the AHR); *b*) inhibition of fatty acid oxidation; and *c*) an increase in peripheral fat mobilization, hepatic steatosis, and *Cyp1a2* RNA levels (Lee et al. 2010). However, we observed that Western diet had relatively little differential effect on the mRNA levels of the *Cyp1* family of genes [see Supplemental Material, Table S5 (http://dx.doi.org/10.1289/ehp.1205003)]. These results suggest that the effect on liver metabolism by Western diet via AHR signaling is not predominately through the *Cyp1* xenobiotic pathway (Lahvis et al. 2000) and that some component(s) in Western diet affects the AHR to directly and/or indirectly induce nonxenobiotic signaling, which in turn, causes large changes in liver metabolism that can affect the propensity for obesity.

*Caloric intake and metabolism.* Although we found no significant differences in caloric intake between regular and Western diets for either mouse strain (Figure 1F), several studies have shown that some mouse strains, when subjected to a high-fat diet, consume fewer kilocalories than control mice on low-fat diets. For example, six of seven mouse strains, including C57BL/6J and DBA/2 (low-affinity AHR), showed an increased adiposity on a high-fat diet but consumed fewer kilocalories than control mice during a 7-week regimen (West et al. 1992). In another study, C57BL/6 mice fed high-fat diets gained significantly more mass per kilocalorie consumed than did the control group on low-fat chow (Black et al. 1998). The interpretations of these studies were that as body adiposity increases, metabolic regulatory signals are activated to decrease energy intake to limit further obesity (West et al. 1992) and that multiple metabolic pathways are suppressed in obese mice and limit energy expenditure (Black et al. 1998). We suggest that may have a role in these metabolic mechanisms regulating obesity.

*The AHR, other nuclear receptors, and obesity.* Understanding the regulatory pathways that govern fat synthesis, accumulation, and catabolism are key to understanding obesity, and nuclear receptors are critical components. Many nuclear receptors are sensors and regulators of fat metabolism. The various nuclear receptors involved in fat metabolism participate in extensive cross-regulatory and cross-signaling interactions among each other and with the AHR. We found that gene expression levels of numerous genes encoding nuclear receptors are differentially affected by diet in B6 and B6.D2 mice and that many of the promoters in genes encoding nuclear receptors possess AHR REs (Table 2). Probably the most important are the PPARs, which are stimulated by fatty acids and derivatives that act as ligands to promote lipid synthesis and storage in adipocytes [PPARg (PPAR-γ), 1 AHR RE, 1.61 in B6W/B6R] and to activate oxidation pathways in the liver [PPARa (PPARα), 15 AHR REs, ↓0.66 in B6W/B6.D2W) and in muscle and brown adipocytes (PPARδ) (Evans et al. 2004). We hypothesize that the Western diet–activated AHR in the two *Ahr* mouse strains differentially interacts with the PPAR signaling pathways to cause different severity levels of obesity.

**Table 2:** mRNA levels of genes encoding nuclear receptors are affected by differential AHR signaling via Western vs. regular diets.

No. of AHR REs^a^	Fold change (p ≤ 0.05)						
	Gene	B6W/B6.D2W	B6R/B6.D2R	B6W/B6R	B6.D2W/B6.D2R
Nr1h3 (Lxr)		5								0.85
Nr1h3 (Lxr)		5						0.85		0.72
Nr1i2 (Pxr)		0						1.22		
Nr1i3 (Car)		2								0.74
Nr2c1 (Tr2)		5						0.70		0.79
Nr2f6		21						0.79		0.85
Nr5a2		1						0.81		
Ppara		15		0.66						
Pparg		1						1.61		
Rarb		0		0.82						1.43
Rarb		0								1.32
Rarg		6				1.08				
Rxra		10						0.69		0.67
Rxra		10						0.69		0.67
Rxrb		0								0.77
Rxrg		7				1.22				1.16
Abbreviations: Lxr, liver X receptor; Pxr, pregnane X receptor; Tr2, testicular receptor 2. n = 4 mice/group. aData from Sun et al. 2004.										

*The AHR as a therapeutic approach to obesity.* We have shown that mice with the high-affinity AHR (*Ahr*^b1^) are more susceptible to obesity than mice with the low-affinity AHR (*Ahr*^d^) when fed Western diet. Epidemiological studies need to be carried out to determine whether diverse AHR signaling activities in the human population are associated with obesity. The broad ligand binding capacity of the AHR may allow relatively easy manipulation of AHR signaling via dietary compounds that act as AHR antagonists, such as curcumin (Ciolino et al. 1998) and resveratrol (Beedanagari et al. 2009). In addition, there are available small molecules that act as powerful AHR antagonists, including CH-223191 (Kim et al. 2006; Zhao et al. 2010), 6,2,4-trimethoxyflavone (Murray et al. 2010), and GNF351 (Smith et al. 2011). The regulation of AHR levels and activity by small interfering RNA approaches (Huang et al. 2011) could also prove promising. The many natural and synthesized antagonists suggest the potential for simple preventative and therapeutic antiobesity strategies via the AHR.

## Supplemental Material

(434 KB) PDF

(913 KB) XLS
