# Supplementary material for: Obesity Is Mediated by Differential Aryl Hydrocarbon Receptor Signaling in Mice Fed a Western Diet
Source: Environ Health Perspect. 2012 May 18;120(9):1252–9. doi: 10.1289/ehp.1205003 (PMC3440132; doi:10.1289/ehp.1205003)
Supplement: (434 KB) PDF [file ehp.1205003.s001.pdf]

Supporting Materials

Title: Obesity Is Mediated by Differential Aryl Hydrocarbon Receptor Signaling in Mice Fed A Western Diet

Authors: Joanna S. Kerley-Hamilton, Heidi W. Trask, Christian J. A. Ridley, Eric DuFour, Carol S. Ringelberg, Nilufer Nurinova, Diandra Wong, Karen L. Moodie, Samantha L. Shipman, Jason H. Moore, Murray Korc, Nicholas W. Shworak, Craig R. Tomlinson

Table of Contents **Page**

Title and Authors 1
Table S1. PCR Primers 2
Table S2. QPCR Results 3
Table S3. B6 vs. B6.D2 Body Mass 4
Table S4. B6 vs. B6.D2 Body Mass Percentages 5
Table S5. B6 vs. B6.D2 Differentially Expressed Liver mRNA Levels 6
Table S6. Complete B6 vs. B6.D2 Liver miRNA Gene List 7
Table S7. Obesity-Related miRNA Gene List. References 12

**Table S1. Primer Specifications**		
**Gene**	**Primers**	**Amplicon Base Pair Length**
Cyp2d26	Forward 5'-GCC GTG TCC ACC AGG AAA TCG-3'<br>Reverse 5'-GAT GAA GAA GTC TTG GAA TTT AAT G-3'	182
Sqle	Forward 5'-CTT GCA TCA GCT CCG AAA AGC-3'<br>Reverse 5'-GCA ACC CAA CAG GAC CGG TCA C-3'	77
Ppar α	Forward 5'-GGCTTCTTTCGGCGAACTATT-3'<br>Reverse 5'-CCGATCACACTTGTCGTACAC-3'	54
Mt1	Forward 5'-AAGAGTGAGTTGGGACACCTT-3'<br>Reverse 5'-CCGATCACACTTGTCGTACAC -3'	111
Lcn2	Forward 5'-GGGAAATATGCACAGGTATCCTC-3'<br>Reverse 5'-GCCACTTGCACATTGTAGCTC-3'	51
Hsd3b5	Forward 5'-AGTGCTAAATAGCGTGTTTACCA-3'<br>Reverse 5'-ACTTTTTGTGTAGTGTCTCCCTG-3	152

Table S2. The QPCR Results Confirm the Microarray Results (*p* -value ≤0.05).

Gene	Gene Fold Change for Microarrays vs. QPCR							
	B6W / B6.D2W		B6R / B6.D2R		B6.D2W / B6.D2R		B6W / B6R	
	Microarray	QPCR	Microarray	QPCR	Microarray	QPCR	Microarray	QPCR
Cyp2d26	42.02	5293						
Hsd3b5					0.03	0.0034	0.03	0.0003
Lcn2					32.26	36.00	15.63	22.16
Mt1			0.25	0.14				
Ppara	0.66	0.33						
Sqle					0.14	0.0007	0.21	0.08

B6 = high-affinity AHR mouse strain.
B6.D2 = low-affinity AHR mouse strain.
W = Western diet; R = Regular diet
n = 4 mice per experimental group.

Table S3. Body Mass and Standard Error of the Mean (SEM) of B6 and B6.D2 Mice Fed Regular Chow or Western Chow for 27 Weeks Beginning at 5 Weeks of Age.

Weeks On Diet	B6 Regular Diet		B6.D2 Regular Diet		B6 Western Diet		B6.D2 Western Diet	
	Ave. Mass	SEM	Ave. Mass	SEM	Ave. Mass	SEM	Ave. Mass	SEM
0	19.25	0.25	20.50	0.71	20.38	0.26	20.00	0.50
1	21.75	0.25	22.63	0.60	23.75	0.16	23.13	0.35
2	24.00	0.38	24.38	0.46	25.13	0.23	24.38	0.53
3	25.25	0.25	25.38	0.42	26.88	0.30	25.63	0.80
4	26.13	0.23	26.25	0.49	28.00	0.42	26.25	1.28
5	27.00	0.27	27.25	0.45	30.88	0.40	28.75	0.94
6	27.75	0.31	27.88	0.48	32.50	0.42	30.13	1.03
7	28.25	0.37	28.38	0.56	34.00	0.53	32.13	1.16
8	28.75	0.49	29.00	0.60	35.50	0.46	33.00	1.10
9	29.38	0.50	29.75	0.62	37.50	0.46	34.00	1.30
10	29.88	0.35	30.38	0.60	38.00	0.57	35.25	1.28
11	30.38	0.38	30.63	0.68	38.75	0.70	36.50	1.36
12	30.38	0.38	31.25	0.73	40.00	0.71	37.50	1.39
13	30.88	0.40	32.00	0.53	40.88	0.85	37.63	1.43
14	31.75	0.25	32.00	0.50	41.63	0.80	38.25	1.44
15	32.00	0.46	32.63	0.53	42.75	0.73	40.13	1.68
16	32.13	0.44	33.25	0.65	44.00	0.68	40.25	1.71
17	32.63	0.46	33.63	0.86	45.88	0.69	41.13	1.66
18	33.00	0.53	34.13	0.72	46.00	0.63	41.50	1.63
19	33.63	0.46	34.13	0.67	47.00	0.60	41.50	1.63
20	32.50	0.57	34.88	0.69	46.13	0.90	43.00	1.65
21	32.88	0.35	34.75	0.65	46.88	0.85	41.88	1.30
22	34.00	0.33	34.88	0.83	49.00	0.63	42.25	1.35
23	35.00	0.57	35.88	0.64	49.75	0.62	43.75	1.42
24	35.00	0.42	36.38	0.82	49.00	0.60	44.75	1.39
25	35.75	0.56	36.88	0.79	51.25	0.53	44.50	1.64
26	36.38	0.78	37.25	0.67	51.63	0.46	44.50	1.77
27	36.38	0.73	37.50	0.76	51.38	0.50	44.88	1.88

Ave. = Average.
B6 = high-affinity AHR mouse strain.
B6.D2 = low-affinity AHR mouse strain.
All mice were male.
n = 8 mice per experimental group.

Table S4. B6 Mice Become Significantly More Obese than B6.D2 Mice When Fed a High-Fat Diet.

Weeks On Diet	% Body Mass B6 / B6.D2			
	Regular Diet	*p*-value	Western Diet	*p*-value
0	93.9	0.11779	101.9	0.51763
1	96.1	0.19709	102.7	0.12837
2	98.5	0.53918	103.1	0.21588
3	99.5	0.80184	104.9	0.16492
4	99.5	0.82053	106.7	0.21464
5	99.1	0.64198	107.4	0.05624
6	99.6	0.83041	107.9	0.05030
7	99.6	0.85535	105.8	0.16317
8	99.1	0.75130	107.6	0.05518
9	98.7	0.64432	110.3	0.02339
10	98.4	0.48132	107.8	0.06937
11	99.2	0.75219	106.2	0.16413
12	97.2	0.30226	106.7	0.13098
13	96.5	0.11356	108.6	0.07084
14	99.2	0.66156	108.8	0.05928
15	98.1	0.39065	106.5	0.17431
16	96.6	0.17300	109.3	0.06085
17	97.0	0.32470	111.6	0.01953
18	96.7	0.22941	110.8	0.02169
19	98.5	0.54700	113.3	0.00674
20	93.2	0.01891	107.3	0.11779
21	94.6	0.02330	111.9	0.00628
22	97.5	0.34493	116.0	0.00046
23	97.6	0.32313	113.7	0.00172
24	96.2	0.15916	109.5	0.01372
25	96.9	0.26416	115.2	0.00152
26	97.7	0.40975	116.0	0.00163
27	97.0	0.30261	114.5	0.00476

Values in gray are significantly different at ≤ 0.05.
B6 = high-affinity AHR mouse strain.
B6.D2 = low-affinity AHR mouse strain.
All mice were male and began the diet regimen at 5
n = 8 mice per experimental group.

Supplemental Material Table S5
Differentially Expressed mRNA Levels of All Genes of All Experimental Conditions (p-value ≤ 0.05)

This table is provided as a separate Excel file.

Table S6. Differentially Expressed Mouse miRNA Genes of All Experimental Conditions (p-value ≤0.05).

Transcript Name	Transcript Type	Fold Change			
		B6R/B6D2R	B6W/B6D2W	B6W/B6R	B6D2W/B6D2R
miR-205	miRNA			4.55	5.26
miR-1224	miRNA			2.94	4.17
miR-1274a	miRNA				4.17
miR-1949	miRNA				3.85
miR-200a	miRNA				3.57
miR-429	miRNA			3.23	3.45
miR-1937b	miRNA	2.49			3.33
miR-200b	miRNA			3.70	3.03
miR-155	miRNA			4.00	2.94
miR-212	miRNA	0.52		5.00	2.78
miR-200b-star	miRNA			2.38	2.78
miR-132	miRNA	0.72		4.17	2.56
miR-34a	miRNA			3.23	2.44
miR-486	miRNA				2.44
v11_miR-685	miRNA				2.44
miR-342-5p	miRNA			3.57	2.38
miR-214	miRNA				2.38
miR-342-3p	miRNA			3.03	2.27
miR-181d	miRNA				2.27
miR-149	miRNA			2.04	2.13
miR-181c	miRNA			1.56	2.13
miR-497	miRNA			1.59	1.92
miR-130b	miRNA			2.50	1.89
miR-2133	miRNA		0.48		1.89
miR-676	miRNA			1.54	1.85
miR-690	miRNA	2.08			1.85
miR-125b-3p	miRNA				1.85
miR-206	miRNA			1.92	1.79
miR-106b-star	miRNA				1.69
miR-181b	miRNA			2.04	1.67
miR-181a2	stem-loop		0.78		1.64
miR-200a-star	miRNA				1.64
miR-291b-5p	miRNA				1.64
miR-542-5p	miRNA	0.57		2.22	1.59
miR-3470b	miRNA				1.54
miR-322	miRNA			1.79	1.47
miR-296-3p	miRNA				1.47
miR-92a-star	miRNA				1.47
miR-6922	stem-loop				1.45
miR-181d	stem-loop				1.41
miR-682	stem-loop				1.41
miR-223	miRNA			2.50	1.39
miR-149	stem-loop			1.25	1.39
miR-130b	stem-loop	1.19			1.32

Table 6 (continued)					
	Transcript	Fold Change			
Transcript Name	Type	B6R/B6D2R	B6W/B6D2W	B6W/B6R	B6D2W/B6D2R
miR-6921	stem-loop				1.32
miR-6922	stem-loop				1.32
miR-21451	stem-loop	1.45			1.30
miR-125b1	stem-loop				1.30
miR-191	miRNA				1.30
miR-214	stem-loop				1.28
miR-3470a	miRNA				1.28
miR-181a2	stem-loop		0.79		1.27
miR-136	miRNA				1.27
miR-883b-5p	miRNA				1.27
miR-298	stem-loop		0.80		1.25
miR-1195	stem-loop				1.23
miR-342	stem-loop			1.52	1.22
miR-133a	miRNA				1.19
miR-1191	stem-loop				1.14
miR-28	stem-loop	0.79			0.87
miR-327	stem-loop				0.86
miR-1948	stem-loop				0.85
miR-377	miRNA	0.81		1.15	0.84
miR-21-star	miRNA				0.83
miR-20a	stem-loop		1.16		0.82
miR-30b-star	miRNA				0.82
miR-30c2	stem-loop				0.81
miR-713	miRNA				0.80
miR-665	stem-loop	0.81			0.79
miR-30c2	stem-loop				0.79
miR-741	miRNA				0.79
miR-669f	miRNA				0.78
miR-291a-5p	miRNA				0.78
miR-28-star	miRNA				0.77
miR-2182	miRNA				0.75
miR-194	miRNA				0.74
miR-139--5p	miRNA			0.71	0.71
miR-30a-star	miRNA			0.64	0.71
miR-20a	miRNA	1.28	1.38	0.77	0.71
miR-331-3p	miRNA	0.58			0.71
miR-193	stem-loop				0.69
miR-345-3p	miRNA			0.51	0.68
miR-187	miRNA				0.66
miR-30a	miRNA		1.36	0.72	0.64
miR-15a	miRNA				0.64
miR-30c2-star	miRNA				0.60
let7g-star	miRNA				0.57
miR-92a	miRNA	0.82		0.60	0.57
miR-101a	miRNA				0.56
miR-101b	miRNA		1.39		0.56
miR-184	miRNA				0.56
miR-467d-star	miRNA				0.56
miR-29c	miRNA		1.40		0.55

Table 6 (continued)					
	Transcript	Fold Change			
Transcript Name	Type	B6R/B6D2R	B6W/B6D2W	B6W/B6R	B6D2W/B6D2R
miR-7a	miRNA				0.55
miR-22-star	miRNA				0.55
miR-26b	miRNA				0.55
miR-770-5p	miRNA				0.55
miR-345-5p	miRNA				0.53
miR-592	miRNA		1.24		0.52
miR-148a	miRNA				0.48
miR-193	miRNA				0.48
miR-127	miRNA				0.44
miR-1948	miRNA	0.68	0.78	0.31	0.27
miR-146b	miRNA			3.85	
miR-214-star	miRNA			2.50	
miR-199b-star	miRNA			2.17	
miR-301a	miRNA			2.00	
miR-27a-star	miRNA			1.96	
miR-34c	miRNA			1.92	
let7e	miRNA	0.58		1.72	
miR-199a-5p	miRNA			1.61	
miR-466j	miRNA			1.45	
miR-484	miRNA			1.45	
miR-200b	stem-loop			1.43	
miR-1188	miRNA			1.39	
miR-27b-star	miRNA			1.39	
miR-3474	miRNA			1.39	
miR-350	stem-loop			1.39	
let7i-star	miRNA			1.37	
miR-23a	stem-loop		1.17	1.35	
miR-450b-3p	miRNA			1.35	
miR-466a-3p	miRNA			1.35	
miR-669e	miRNA			1.35	
miR-467c	miRNA			1.33	
miR-465a-3p	miRNA		1.20	1.32	
miR-382	miRNA		1.32	1.30	
miR-466b3-3p	miRNA			1.28	
miR-582	stem-loop	0.84	1.14	1.27	
miR-124	miRNA			1.27	
miR-23a	stem-loop			1.27	
miR-146a	stem-loop		1.21	1.23	
let7d	miRNA	0.78		1.23	
miR-1274a	stem-loop	0.86		1.22	
miR-29b2	stem-loop	0.83		1.22	
miR-467d	miRNA		1.16	1.20	
miR-125b1	stem-loop			1.19	
let7i	stem-loop			1.18	
miR-183-star	miRNA			1.18	
miR-34a	stem-loop	0.84		1.16	
miR-27a	stem-loop			1.15	
miR-27b	stem-loop			1.14	

Table 6 (continued)					
	Transcript	Fold Change			
Transcript Name	Type	B6R/B6D2R	B6W/B6D2W	B6W/B6R	B6D2W/B6D2R
miR-710	stem-loop			1.12	
miR-3470b	stem-loop	0.81		1.11	
miR-219	miRNA			0.90	
miR-30d	miRNA			0.85	
miR-22	miRNA	1.32		0.79	
miR-22	stem-loop			0.79	
miR-299-star	miRNA			0.79	
miR-488-star	miRNA	1.33		0.75	
v11_miR-197	miRNA	1.44		0.73	
miR-2146	miRNA		0.67	0.71	
miR-2861	miRNA			0.66	
miR-1939	miRNA			0.65	
miR-714	miRNA			0.63	
miR-762	miRNA			0.58	
miR-1944	miRNA			0.46	
miR-20b	miRNA		2.07		
miR-140	miRNA		2.02		
miR-125b-star	miRNA		1.80		
miR-30e	miRNA		1.75		
miR-106b	miRNA	1.45	1.72		
miR-135a-star	miRNA		1.56		
miR-503-star	miRNA		1.34		
miR-16	miRNA		1.30		
miR-27a	miRNA		1.25		
miR-669l	stem-loop		1.16		
miR-879	miRNA		1.13		
miR-466d	stem-loop	1.18	1.11		
miR-299	stem-loop	0.87	0.91		
miR-692	miRNA		0.86		
miR-485	stem-loop		0.86		
miR-345	stem-loop		0.86		
miR-874	stem-loop		0.85		
miR-6803	stem-loop		0.83		
miR-466c	stem-loop		0.82		
miR-669h	stem-loop		0.79		
miR-101a	stem-loop		0.77		
miR-1946a	miRNA		0.73		
miR-320	stem-loop		0.69		
miR-465c1	stem-loop		0.41		
miR-1839-3p	miRNA	3.56			
miR-2183	miRNA	1.70			
miR-93	stem-loop	1.36			
miR-489	miRNA	1.19			
miR-669j	miRNA	1.14			
miR-26a1	stem-loop	1.12			
miR-669h	stem-loop	1.10			
miR-466b3	stem-loop	0.93			
miR-325	stem-loop	0.92			

Table 6 (continued)					
	Transcript	Fold Change			
Transcript Name	Type	B6R/B6D2R	B6W/B6D2W	B6W/B6R	B6D2W/B6D2R
miR-469	stem-loop	0.89			
miR-326	stem-loop	0.89			
miR-669f	stem-loop	0.86			
miR-449b	miRNA	0.84			
miR-712-star	miRNA	0.84			
miR-330	stem-loop	0.81			
miR-151-5p	miRNA	0.80			
miR-297a6	stem-loop	0.78			
miR-1898	miRNA	0.78			
let7b	miRNA	0.77			
miR-361	miRNA	0.74			
miR-468	miRNA	0.70			
miR-1941-5p	miRNA	0.68			
miR-1964	miRNA	0.59			
miR-466f1	stem-loop	0.56			
miR-532-3p	miRNA	0.55			
miR-671-3p	miRNA	0.53			
miR-98	miRNA	0.47			

B6 = high-affinity AHR mouse strain.
B6.D2 = low-affinity AHR mouse strain.
W = Western diet; R = Regular diet.
n = 4 mice per experimental group.

Table S7. Differentially Expressed miRNA Genes (≥ ±2.0 fold) from Liver of B6 vs. B6.D2 Male Mice Fed Regular or High-Fat (Western) Chow for 27 Weeks Beginning at 5 Weeks of Age (*p*-value ≤0.05).

Mouse miRNA Transcript	Transcript Form	Experimental Comparison /				Relevance to AHR and Obesity	References
		B6R / B6D2R	B6W / B6D2W	B6W / B6R	B6D2W / B6D2R		
miR-20b	miRNA		2.07			Resveratrol, AHR ligand	[1]
miR-140	miRNA		2.02			described in this paper	
miR-2133	miRNA		0.48		1.89	described in this paper	
miR-465c1	stem-loop		0.41			described in this paper	
miR-1839-3p	miRNA	3.56				described in this paper	
miR-690	miRNA	2.08			1.85	described in this paper	
miR-98	miRNA	0.47				described in this paper	
miR-212	miRNA	0.52		5.00	2.78	Insulin resistance	[2]
miR-205	miRNA			4.55	5.26	described in this paper	
miR-132	miRNA	0.72		4.17	2.56	Obesity, NAFLD, Nutrtional stress	[3-5]
miR-155	miRNA			4.00	2.94	Hepatocarcinogenesis	[6]
miR-146b	miRNA			3.85		described in this paper	
miR-200b	miRNA			3.70	3.03	NAFLD	[7]
miR-342-5p	miRNA			3.57	2.38	described in this paper	
miR-34a	miRNA			3.23	2.44	NAFLD	[7]
miR-429	miRNA			3.23	3.45	described in this paper	
miR-342-3p	miRNA			3.03	2.27	described in this paper	
miR-1224	miRNA			2.94	4.17	described in this paper	
miR-130b	miRNA			2.50	1.89	Adipogenesis, PPARγ	[8]
miR-214*	miRNA			2.50		described in this paper	
miR-223	miRNA			2.50	1.39	Leptin, muscle mass	[9]
miR-200b*	miRNA			2.38	2.78	described in this paper	
miR-542-5p	miRNA	0.57		2.22	1.59	described in this paper	
miR-199b*	miRNA			2.17		described in this paper	
miR-149	miRNA			2.04	2.13	described in this paper	
miR-181b	miRNA			2.04	1.67	described in this paper	
miR-301a	miRNA			2.00		described in this paper	
miR-1948	miRNA	0.68	0.78	0.31	0.27	described in this paper	
miR-1274a	miRNA				4.17	described in this paper	
miR-1949	miRNA				3.85	described in this paper	
miR-200a	miRNA				3.57	NAFLD	[10]
miR-486	miRNA				2.44	described in this paper	
miR-214	miRNA				2.38	described in this paper	
miR-181d	miRNA				2.27	Intracellular lipid droplet formation	[11]
miR-181c	miRNA			1.56	2.13	described in this paper	
miR-148a	miRNA				0.48	Liver injury	[12]
miR-193	miRNA				0.48	described in this paper	
miR-127	miRNA				0.44	described in this paper	
miR-29c	miRNA		1.40		0.55	NAFLD	[7]
miR-30c2	stem-loop				0.81	Adipogenesis	[13]
miR-30a	miRNA		1.36	0.72	0.64	Adipogenesis	[13]
miR-30d	miRNA			0.85		Adipogenesis	[13]
miR-30e	miRNA		1.75			Adipogenesis	[13]

B6 = high-affinity AHR mouse strain.
B6.D2 = low-affinity AHR mouse strain.
W = Western diet; R = Regular diet.
NAFLD, non-alcholic fatty liver disease
n = 4 mice per experimental group.

References

1 Dhar S, Hicks C, & Levenson AS (2011) Resveratrol and prostate cancer: Promising role for microRNAs. Mol. Nutr. Food Res. 55(8):1219-1229.
2 Pulakat L, Aroor A, Gul R, & Sowers J (2012) Cardiac Insulin Resistance and MicroRNA Modulators. Experimental Diabetes Research 2012.
3 Heneghan HM, et al.J (2011) Differential miRNA Expression in Omental Adipose Tissue and in the Circulation of Obese Patients Identifies Novel Metabolic Biomarkers. J Clinical Endocrin. Metab. 96(5):E846-E8[illegible]
4 Estep M, et al. (2010) Differential expression of miRNAs in the visceral adipose tissue of patients with non-alcoholic fatty liver disease. Alimentary Pharmacology & Therapeutics 32(3):487-497.
5 Strum JC, et al. (2009) MicroRNA 132 Regulates Nutritional Stress-Induced Chemokine Production through Repression of SirT1. Molecular Endocrinology 23(11):1876-1884.
6 Wang B, et al. (2009) Role of microRNA-155 at early stages of hepatocarcinogenesis induced by choline-deficient and amino acid-defined diet in C57BL/6 mice. Hepatology 50(4):1152-1161.
7 Pogribny IP, et al. (2010) Hepatic miRNAs miR-29c, miR-34a, miR-155, and miR-200b is associated with strain-specific susceptibility to dietary nonalcoholic steatohepatitis in mice. Lab Invest. 90:1437.
8 Lee EK, et al. (2011) miR-130 Suppresses Adipogenesis by Inhibiting Peroxisome Proliferator-Activated Receptor {gamma} Expression. Mol. Cell. Biol. 31(4):626-638.
9 Hamrick MW, et al. (2010) The adipokine leptin increases skeletal muscle mass and significantly alters skeletal muscle miRNA expression profile in aged mice. BBRC 400(3):379-383.
10 Alisi A, et al. (2011) Mirnome analysis reveals novel molecular determinants in the pathogenesis of diet-induced nonalcoholic fatty liver disease. Lab Invest. 91(2):283-293.
11 Whittaker R, et al. (2010) Identification of MicroRNAs That Control Lipid Droplet Formation and Growth in Hepatocytes via High-Content Screening. Journal of Biomolecular Screening 15(7):798-805.
12 Farid WRR, et al. (2011) Hepatocyte-derived micrornas as serum biomarker of hepatic injury and rejection after liver transplantation. Liver Transpl. 18(3):290-297
13 Zaragosi L-E, et al. (2011) Small RNA sequencing reveals miR-642a-3p as a novel adipocyte-specific microRNA and miR-30 as a key regulator of human adipogenesis. Genome Biology 12(7):R64.
